# The language of the immune system: Part 1: Cytokine conversations and conversationalists

**DOI:** 10.1093/immhor/vlag036

**Published:** 2026-08-04

**Authors:** Ken Steven Rosenthal

**Affiliations:** Augusta University/University of Georgia Medical Partnership, Dept. of Basic Sciences, Athens, GA, United States; University of Georgia College of Veterinary Medicine, Dept. of Infectious Diseases, Athens, GA, United States; NE Ohio Medical University, Rootstown, OH, United States

**Keywords:** acute phase cytokines, alarmins, chemokines, cytokines, T cell cytokines

## Abstract

The immune system is always acting to protect the body from the microbial world, especially at the openings of the body but also helps to maintain homeostasis. Normal functions include self-regulation and regeneration, facilitating the maintenance of organs, skin and mucosal membranes, protecting the openings of the body, providing surveillance for abnormal and cancer cells and eliminating molecular and cellular debris. In addition to status quo maintenance functions, the immune system responds to damage, disruption or danger when challenged by trauma, infection, cancer, autoimmunity and other diseases. The responses are communicated and coordinated to other cells, tissues, organs, and systems through cell-cell interactions, with different soluble molecules including cytokines. Cytokines are the language of the immune system and define the progression of the subsequent immune response. Each cytokine may elicit its own responses but by acting together in a **conversation** they communicate and coordinate the intended actions to maintain the status quo or respond to challenge. These conversations include regulation/tolerance, alarmins, acute phase cytokines, Th1, Th2, Th17, and Th22. The cytokine conversations, their conversationalists (the cells eliciting and responding to cytokines), and the basic responses of the immune system will be discussed. An easy way to remember the conversations is provided.

## Introduction

In addition to responding to infectious challenges, the immune system is constantly communicating with and facilitating the proper function of all tissue-organ systems of the body. Its routine tasks include self-regulation and regeneration, facilitating the maintenance of organs, skin and mucosal membranes, protecting the openings of the body, providing surveillance for abnormal and cancer cells and eliminating molecular and cellular debris. When posed with damage, disruption, or danger from trauma, infection, cancer, autoimmunity or other diseases, the immune system rises and responds to the challenge to protect and resolve the problem. These responses require production of new and different cells and proteins, changes in metabolism as well as recruitment, mobilization, and activation of appropriate cells to resolve the problem and then restore the status quo and homeostasis. All of this requires a great amount of energy, coordination, and especially communication.

The immune system communicates and coordinates local and systemic responses with soluble molecules (ions, metabolites, lipids [prostaglandins and leukotrienes], and proteins) and cell-cell ligand-receptor interactions. **Cytokines, interferons, and chemokines are small hormone-like proteins that provide the vocabulary for communication between immune and other cells.** Heretofore, they will all be referred to as cytokines. **Interferons** got their name because they activate cellular processes that “interfere” with virus production, but they have other influences on cells and the immune response. The primary functions of **chemokines** are to recruit and direct the movement of cells, especially white blood cells.

**Figure 1:**
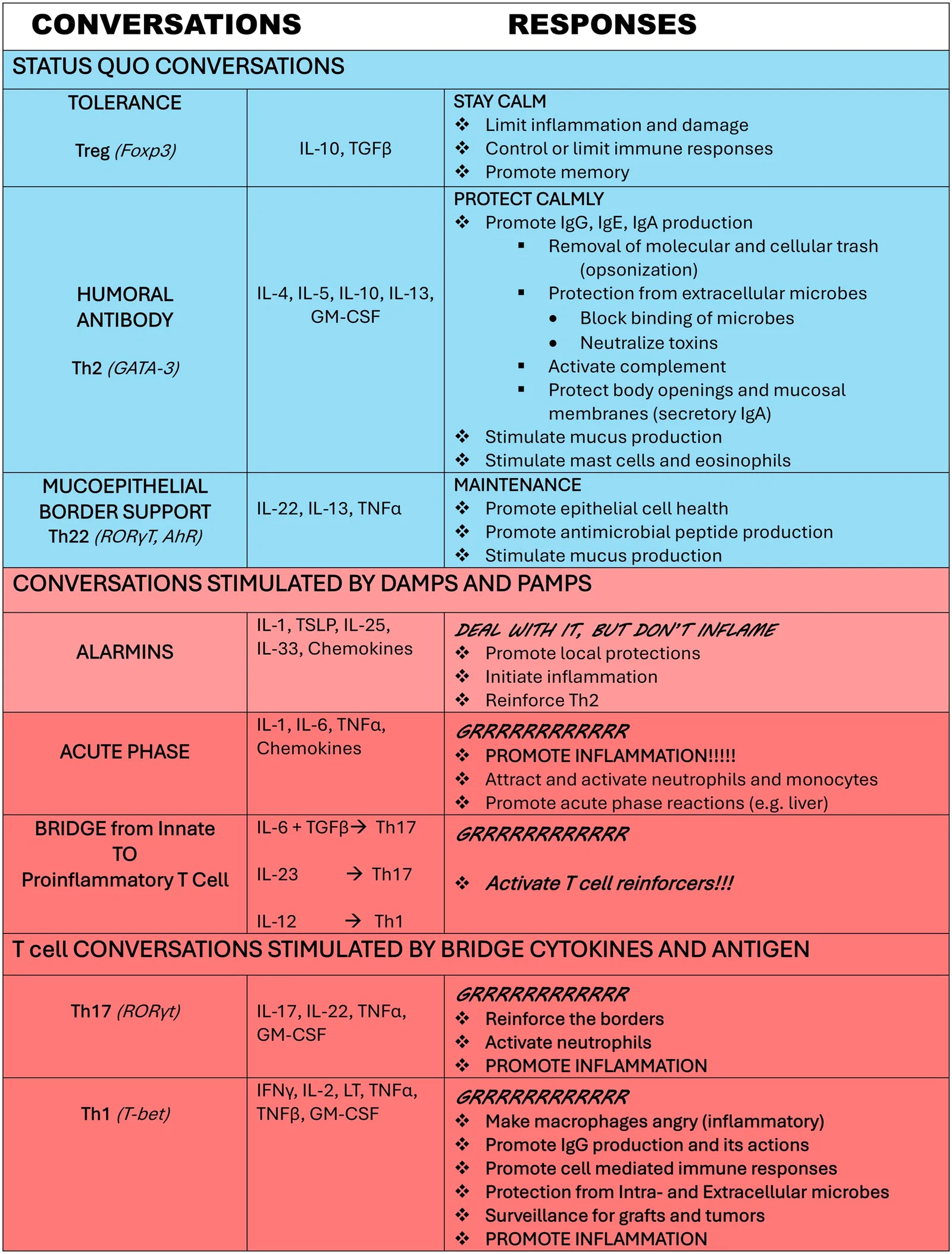
Cytokine conversations and their responses. A useful way to learn and remember the cytokine conversations is to voice them out loud with the emotion that reflects the responses that they elicit, eg calm and quiet for conversations that maintain the status quo or regulation and loud and angry (GRRRRRRRRR) for conversations that elicit inflammation. The conversations in blue are calming and promote status quo and tolerance functions whereas the conversations in pink and red are angry and elicit or reinforce inflammation and related responses. Transcription factors that activate and determine T cell responses are inserted next to the Th in italics.

Although each of the cytokines has its own nuanced activities, they also act with other cytokines as a **conversation** to communicate and coordinate the intended response of cells, tissues and organs. As with the English language, a cytokine may elicit different responses, just as a word may have different pronunciations or meanings depending upon context, for example, lead, as in leader, or the lead metal. Conversations with other cytokines provide the context to focus on the response and subsequent consequence. For example, the interleukin (IL)-1 cytokine has many different activities, including stimulating cell growth, but in an acute phase conversation with IL-6 and tumor necrosis factor (TNF)**-**α, it promotes inflammation. The characteristics of several of the key cytokines and their cytokine conversations are summarized in Table 1 and Fig. 1.

**Table 1:** The cytokine vocabulary.^a^

Cytokine	Major sources^b^	Major target^b^	Primary functions^b^
**INNATE and ACUTE-PHASE CONVERSATIONS**			
Type 1 IFN: IFN-α, IFN-β, IFN-ε	pDCs, MPs, fibroblasts, epithelial cells	Virally infected cells, tumor cells, NK cells	Induction of antiviral state; activation of NK cells, enhancement of cell-mediated immunity, B cell activation
IL-1α, IL-1β	MPs, DCs, fibroblasts, epithelial cells, endothelial cells	T cells, B cells, PMNs, MPs	Promotion of inflammatory and acute-phase responses, fever, activation of lymphocytes and MPs, etc.
TNF-α	Similar to IL-1	MPs, T cells, NK cells, epithelial, PMN, mast, and many other cells	Promotion of inflammatory and acute-phase responses, fever, antitumor, wasting (cachexia), sepsis, activation of mast cell release of histamine, activation of neutrophils, monocytes and MPs and other cells, promotes release of IL-1, other cytokines and chemokines, etc.
IL-6	DCs, MPs, T and B cells, fibroblasts, epithelial cells, endothelial cells	T and B cells, hepatocytes, other cells	Stimulation of acute-phase and inflammatory responses, T- and B- cell growth and development, etc.
**BRIDGE to PROINFLAMMATORY T CELL CONVERSATIONS**			
IL-12, IL-23 (share a p40 subunit)	DCs, MPs	NK cells, CD4 Th1, Th17 cells	Activation of T-cell-mediated and inflammatory responses IL-12: promotes IFN-γ (Th1) production IL-23: promotes IL-17 (Th17) production
**GROWTH and DIFFERENTATION**			
Colony-stimulating factors (eg G-CSF, GM-CSF)	T cells, stromal cells	Stem cells	Growth and differentiation of specific cell types, hematopoiesis
IL-3	CD4 T cells and other cells,	Hematopoietic stem cells	Hematopoiesis, growth of diverse types of cells
IL-7	Bone marrow, stroma	Stem cells	Growth and differentiation of lymphoid stem cells
**PROINFLAMMATORY Th1 and Th17 CONVERSATIONS**			
IL-2	CD4 Th0, Th1 cells	T cells, Tregs, B cells, NK cells	T- and B-cell growth, Treg activation, NK activation
IFN-γ	CD4 Th1 cells, NK cells, ILC1	MPs, DCs, T cells, B cells	Activation of macrophage, inflammation, Th1, IgG class switch, inhibition of Th2 and Th17 responses
TNF-β (lymphotoxin)	CD4 Th1 cells	PMN, tumors	Tumor killing, activation of PMN, endothelial activation
IL-17	CD4 Th17 cells, ILC3	PMN, MPs, epithelial, endothelial, and fibroblast cells;	Inflammation, antimicrobial peptide production, activation of neutrophils
IL-22	CD4 Th17 cells, Th22, ILC3	Epithelial cells	Growth and repair of epithelial cells; antibacterial peptide production
GM-CSF	Th1 and Th17 cells	Myeloid cells	Expansion and activation of neutrophils and monocytes
**Th2 ANTIBODY AND HUMORAL CONVERSATIONS**			
IL-4	CD4 T cells (Th0, Th2), ILC2	B and T cells, mast cells	T- and B-cell growth; IgG, IgA, and IgE production; reinforce Th2 responses, stimulates mast cells
IL-5	CD4 Th2 cells, ILC2	B cells, eosinophils	B-cell growth and differentiation; IgG, IgA, and IgE production; eosinophil production; allergic responses
IL-10	CD4 Th2, ILCreg, Treg, B cells	B cells, other cells	B-cell growth, inhibition of Th1 response and inflammation, regulation
IL-13	CD4 Th2 and Th22	Goblet, mast cells, and epithelial cells	Mucus production, stimulates mast cells, allergy symptoms, anti-parasitic worm defense
GM-CSF	Th1 and Th17 cells	Myeloid cells	Expansion of neutrophils and monocytes
**REGULATORY CONVERSATIONS**			
TGF-β	CD4 Treg, ILCreg	B cells, T cells, MPs, other cells	Immunosuppression of B, T, and NK cells, MPs; promotion of oral tolerance, wound healing, fibrosis, IgA production
IL-10	See above		
**CHEMOKINES**			
α-Chemokines: CXC chemokines, 2 cysteines separated by 1 amino acid (IL-8; IP-10; GRO-α, GRO-β, GRO-γ)	Many cells	PMN, T cells, MPs	Chemotaxis, activation
β-Chemokines: CC chemokines, 2 adjacent cysteines (MCP-1; MIP-α; MIP-β; RANTES)	Many cells	T cells, MPs, basophils	Chemotaxis, activation

a Adapted from Table 6.1, Medical Microbiology, 10e. Murray, Rosenthal and Pfaller, Elsevier 2025.

b Not all inclusive.

CD, Cluster of differentiation; DCs, dendritic cells; MPs, macrophages; GM-CSF, granulocyte-macrophage colony-stimulating factor; GRO-γ, growth-related oncogene-γ; IFN-α, -β, -γ, interferon-α, -β, -γ; Ig, immunoglobulin; IL, interleukin; ILC, innate lymphoid cells; ILCreg, regulatory innate lymphoid cell; IP, interferon-α protein; MCP, monocyte chemoattractant protein; MIP, macrophage inflammatory protein; NK, natural killer cells; PMN, polymorphonuclear leukocyte; RANTES, regulated on activation, normal T expressed and secreted; TGF-β, transforming growth factor-β; Th, T helper (cell); TNF, tumor necrosis factor; Treg, all regulatory T cells.

*A useful way to learn and remember the cytokine conversations is to voice them out loud with the emotion that reflects the responses that they elicit, for example calm and quiet for conversations that maintain the status quo or regulation and loud and angry for conversations that elicit inflammation, as indicted by the cues in* *Fig. 1*.

Within the last 10 yr, the roles and importance of cytokines in communicating and coordinating the actions of the immune system have become more important to understanding and treating infectious, inflammatory and immunological diseases.^1^^,^^2^ This review (Part 1) provides an update and perspective for students, immunology and non-immunology faculty, and others by introducing the conversationalists (cells), their cytokine vocabularies and conversations, and the triggers that promote the conversations. References provide reviews with good figures that expand upon the concepts. *As mentioned earlier, the emotions indicated in* *Fig. 1 provide a useful means of teaching and learning the consequences of the cytokine conversations.*

When conversations get out of control and become excessively loud, inappropriate, disorganized, or unending (chronic) they can be disruptive. For cytokine conversations, this can promote disorder, excessive inflammation, dysfunction, self-abuse or chronicity and disease. Knowing which cytokine words and conversations are driving the disease allows pharmacological treatment that can tone down the entire system (eg corticosteroids), inactivate key cytokine words (inhibitory monoclonal antibodies) or prevent the cytokine response (Janus tyrosine kinase inhibitors).^3^

## The conversationalists: cells of the immune system

The immune system can be divided into the innate subsystem and the adaptive (antigen responsive) subsystem. The **innate subsystem** includes barriers, soluble molecules, epithelial cells, granulocytes, phagocytes, natural killer (NK) and other cells that are pre-programmed to respond to molecules released during tissue damage **(damage associated molecular patterns [DAMPS])** and molecular patterns of pathogenic microbes **(pathogen associated molecular patterns [PAMPS]).** Innate responses are strengthened by increasing the number of cells, increasing their activity and extending their lifespan. The lifetime of innate responses can be extended by epigenetic, transcriptional, and metabolic changes in a process termed trained immunity.^4^ The **adaptive subsystem** consists of B and T lymphocytes (B cells and T cells) their progeny (memory and plasma cells) and secreted antibodies. Antibody and cell surface B cell receptors (BCR) for B lymphocytes and cell surface T cell receptors (TCR) for T cells recognize specific molecular structures (called **epitopes**). The epitopes may be part of larger molecules, **antigens**, which may contain multiple epitopes. Lymphocytes are also responsive to PAMPs and DAMPs. Adaptive responses are strengthened by clonal expansion of antigen-specific lymphocytes. Unlike cells of the innate subsystem, B and T lymphocytes can differentiate into long lived memory cells for faster and stronger recall of an antigen response in the future.^5^ The subsystems are intertwined and innate cells initiate, support and regulate lymphocyte responses, while lymphocyte responses support and regulate innate responses and both subsystems produce and are responsive to cytokines.

Immune responses often begin at the barriers and openings of the body in response to everyday challenges from microbiota and minor traumas, for example sprains, cuts, scratches, bumps, bruises, etc. As such, many cytokine conversations are initiated by **epithelial cells** of the respiratory and gastrointestinal tracts and **keratinocytes** of skin. Underneath these barriers are a mixture of other cells that provide support and respond to deeper challenges. These include dendritic cells (**DCs**), macrophages (**MPs**), innate lymphoid cells (**ILCs**), and memory lymphocytes, especially memory T cells. A specialized version of a DC in the epidermis of skin is called a Langerhans cell. When needed, other cells, such as neutrophils, monocytes, or eosinophils, are recruited to help deal with a challenge.

### Epithelial cells

Epithelial cells, keratinocytes and other cells have sensors (receptors) for microbes that recognize microbial PAMPs and DAMPs released upon tissue damage (see “Conversation Triggers”).^6^^,^^7^ One way they respond to danger is by activation and assembly of **inflammasomes,** which are large, multi-subunit proteases that initiate local inflammation through proteolytic activation and release of the **inflammatory cytokines, IL-1β and IL-18.** In addition, these cells release **alarmins and chemokines** to initiate and guide local inflammation.^8^ Chemokines recruit cells, such as neutrophils and monocytes, to the affected site^8–10^ (Tables 1 and 2).

**Table 2:** Key conversations associated with cells, tissues, responses and diseases.

Cell, Tissue, Disease, etc.	Conversations	Key Cytokine(s)^a^
**Mucus production**	Status quo, Th2^b^	IL-4, IL-5, IL-13
**Mast cells & Eosinophils**	Th2	IL-4, IL-5, IL-33, TSLP
**Neutrophils**	Acute phase; Th17	IL-17, TNF-α, GM-CSF
**Inflammatory Macrophages and DCs**	Acute phase Bridge cytokines Th1	IFN-γ, TNF-α TNF-α, IL-1, IL-6 IL-23, IL-12
**Skin**	Th22	IL-22
**Itch**		IL-31
**Inflammation**	Acute phase; C′; Th17; Th1	TNF-α, IL-1, IL-6; IL-17; IFN-γ
**Sepsis**	Acute phase	TNF-α, IL-1, IL-6
**Allergies**	Th2	IL-4, IL-5, IL-13
**Asthma**	Th2	IL-4, IL-5, IL-13
**Psoriasis**	Th22; Th17	IL-22; IL-23, IL-17, TNF-α
**Bacterial and fungal infections**	Acute phase; Th 17; Th1	IL-1, IL-6, TNF-α; IL-17; IFN-γ
**Virus infections**	Type 1 IFNs; Th1	IFN-α, IFN-ε, IFN-γ
**Parasitic worm infections**	Th2	IL-4, IL-5, IL-13

a Other cytokines are also involved.

b Not all inclusive.

C′, complement; IFN, interferon; IL, interleukin; Th, T helper response; TNF, tumor necrosis factor; TSLP, Thymic Stromal Lymphopoietin.

### Myeloid cells

Myeloid cells include important **antigen presenting cells** (APCs), such as DCs, monocytes and macrophages (MPs), and granulocytes, such as neutrophils and eosinophils. These cells are responsive to PAMPs, DAMPs and cytokines and are **phagocytic.** 

**DCs** and **Langerhans cells** have multiple long arms (dendrites) that stick out through the epithelium and constantly taste the antigenic milieu. Immature and local DCs usually promote immunological tolerance to these antigens and regulation to prevent responses to commonplace proteins and minimize responses to minor traumas.^11–14^ When DCs sense danger following tissue damage or the presence of infecting microbes, they release a molecular conversation of cytokine warnings (see below), mature, and migrate to lymph nodes taking with them the phagocytized antigenic evidence of the danger. While en route, the maturing DCs proteolytically process ingested proteins and display their peptides on the cell surface within clefts of the **major histocompatibility antigen (MHC) I and MHC II proteins**.^15^ DCs localize to the T cell zone within the lymph node where they can encounter circulating naive T cells. When a T cell recognizes one of the antigenic peptide-MHC complexes displayed on the DC surface, the DC wraps its dendrites around the T cell, provides activating receptor-ligand hugs, and the necessary cytokine conversation to activate and coax the antigen-specific T cell to elicit the necessary response. DCs are the primary cell type to initiate a new immune response in naive T cells. ***D*Cs *d*irect the response of the *T* cell so that the *T* cell can *t*ell other cells what to do in response to the challenge.** 

**Plasmacytoid DCs (pDCs)** are a special type of DC that are major responders to infections, especially viral infections.^16^ Upon activation by PAMPRs, they produce large amounts of type I interferons and other cytokines and become potent APCs.

**MPs** (big eaters) can have different identities depending upon their environment or stimuli. **Monocytes** are precursors of MPs that can be recruited from blood to mature where reinforcements are needed.^17^ Tissue resident MPs include alveolar MPs (lung), Kupffer cells (liver), and microglial cells (brain). The primary functions of tissue resident MPs are to phagocytize and eliminate molecular, particulate and cellular debris, help maintain the function of other cells and promote homeostasis (status quo).^18^ Tattoos depend upon skin MPs to phagocytize and retain ink particles.^19^ Phagocytosis is facilitated by certain molecules, called opsonins, that bind to the debris and to receptors for these molecules on MPs. Upon microbial challenge, local MPs recruit neutrophils, monocytes and other cells from the blood by releasing acute phase cytokine and chemokine conversations. PAMPs, inflammatory cytokines and T cells stimulate monocytes and MPs and make them angry (Grrrrrrrrrrrrrrrrr) enhancing their antimicrobial functions as **pro-inflammatory MPs (often termed M1).** Like DCs, MPs are APCs and can manipulate T cell function with cytokines. MPs can also switch to become **anti-inflammatory,** also termed **M2 MPs. M2 MPs** are functionally divided into subtypes (M2 _(a-d)_) which provide support for wound healing, angiogenesis, fibrosis, tissue remodeling and other actions.^20^^,^^21^

**Neutrophils** are short-lived granulocytes that are antimicrobial and promote inflammation.^22^ They are recruited to sites of infection and inflammation and activated by proinflammatory cytokine conversations and T cells.^23^ In addition to killing phagocytized microbes, these cells release enzymes, antimicrobial molecules, cytokines and upon lysis they release sticky neutrophil extracellular traps (NETS) composed of DNA and cytolytic proteins to trap and kill microbes.^24^

**Eosinophils** are cousins of neutrophils that also contain granules filled with inflammatory molecules and produce inflammatory cytokines, but these cells respond to different cytokine conversations.^25^^,^^26^  **Basophils** and **mast cells** are also granulocytes. Eosinophils, mast cells and basophils are important defenses against intestinal worm infestations and effector cells of allergic and asthmatic responses.

### Lymphoid cells

Lymphoid cells consist of T cells, B cells, and innate lymphoid cells (ILCs). The latter group includes NK cells. T and B cells express antigen receptor molecules whereas ILCs do not.

### T cells

T cell precursors are generated in the bone marrow and then educated and selected in the thymus, hence the “T.” T cells are defined by the expression of a TCR and the CD3 molecules.^27^ T cells leave the thymus, enter the blood and home to lymph nodes, spleen and the gastrointestinal system. Upon activation, many of the T cells enter the blood and home to the site of inflammation or infection in response to chemokines. T cells include unconventional and conventional T cells.

**Unconventional T cells** have αβ TCRs that recognize non-peptide ligands bound to analogues of MHC molecules or a γδ TCR.^28^^,^^29^ They include **natural killer T cells (NKT)** that recognize glycolipids bound to CD1d; **mucosal-associated invariant T (MAIT)** cells that recognize microbial vitamin B derivatives on MR-1 molecules; and **γδ T cells** that recognize small, organic based pyrophosphate containing molecules and other molecules. These cells can initiate or reinforce immune responses with cytokine conversations similar to CD4 T helper cells.

**Conventional T cells** express one of an almost infinite number of different cell surface αβ **TCR** molecules. These TCRs recognize specific antigenic peptide-MHC complexes. A CD4 or CD8 co-receptor molecule interacts with MHC II or MHC I proteins, respectively.

Most **CD4 T cells** are helper T cells mediating responses with defined cytokine conversations and receptor-ligand hugs but can also be cytotoxic. **CD8 T cells** are primarily cytotoxic T cells but can also release defined cytokine conversations similar to CD4 T cells.^30^ Activation and determination of T cell cytokine conversations are initially directed by antigen presentation and cytokines generated by DCs, and later by MPs and other cells. The T cell cytokine conversation is determined by specific transcription factors (see Fig. 1). Following activation, T cells can become **effector or memory T cells**.

Activation of naive T cells requires at least 2 commitment signals, one from the TCR and another from the co-receptor, CD28. Activated APCs express sufficient B7 molecules to bind and fill up inhibitory CTLA-4 receptors and then bind to CD28 to activate the T cell. Adhesion molecules zip up the opposing cell membranes to extend the avidity and length of interaction. Cytokine conversations provide a third signal that will determine the subsequent T cell function.^31^

Upon antigen stimulation, naive T cells (also referred to as Th0 cells) differentiate into subsets defined by specific transcription factors and their cytokine conversations.^31^^,^^32^  **Regulatory T (Treg) cells** are defined by the FOXP3 transcription factor and produce TGFβ, IL-10 and IL-35 to promote tolerance and limit inflammation. **Th2** cells are induced by IL-4, express GATA3 and produce IL-4, IL-5, IL-10, IL-13 and GM-CSF to promote and coordinate B cell class switching to immunoglobulin (Ig)G, IgE and IgA antibodies, mucus production, and mast cell and eosinophil activation. **Th22** cells are induced by IL-6 and TNF-α or by IL-23, express ROR-γT and AhR and express IL-22, IL-13, and TNF-α to promote epithelial cell proliferation, antimicrobial functions and reinforce membrane barriers.

**Th17 and Th1 proinflammatory helper T cells** reinforce innate protections. **Th17** cells reinforce epithelial and neutrophil functions and **Th1** cells reinforce local and systemic protections, promote activation of inflammatory MPs and DCs and antigen-specific responses including cell mediated and IgG antibody responses. Th17 and Th1 responses will be described later in more detail.

**T follicular helper cells (Tfh)** are defined by Bcl6 transcription factor and facilitate germinal center formation of lymph nodes, and B cell growth, survival, differentiation and antibody production.^33^ They do this through production of IL-21 and IL-4 and the interaction of their cell surface CD40 ligand (CD40L) with CD40 on the B cell surface. Tfh also express the transcription factors and cytokines of Th1, Th2 and Th17 to promote IgG, IgE and IgA production.

**Th9** and other CD4 T cell types are named for a dominant cytokine that they produce.

### ILCs

**ILCs** generate cytokine conversations similar to the different helper T cell types and have parallel designations of ILC1 (Th1), ILC2 (Th2), ILC3 (Th17), and ILCreg.^34^^,^^35^ They are stimulated by PAMPs, DAMPs, cytokines and other signals but unlike T cells, do not recognize MHC-peptide complexes nor express CD3 or TCRs. NK cells have similar killing mechanisms as CD8 T cells.

### B cells

**B cells** mature, are selected and become naive B cells in the bone marrow.^36^ The “B” could stand for bone but actually relates to the bursa of Fabricius in birds, where they were first described.^37^ The B cell expresses a **B cell receptor (BCR)** that has the same specificity as its secreted antibody to coordinate antigen specific stimulation and subsequent antibody production. Naive B cells are activated by antigen, cytokines and other signals. The initial antibody produced and released by B cells is IgM. With T cell help, B cells can differentiate to produce IgG, IgE, or IgA to the same antigen.

**Immunoglobulins** are “Y” shaped glycoproteins consisting of at least 2 heavy chains and two light chains held together by disulfide bonds.^38^ The antigen-binding variable regions (Fab) are at the arms of the Y. The trunk region (Fc) interacts with different host molecules and cellular receptors and provide different functions to IgM, IgG, IgE, and IgA. TCRs have an immunoglobulin-like structure. The antigen-binding repertoires of BCRs and TCRs are generated by random recombinations of gene segments within their respective genes. Antibody is further randomized by somatic mutation of the region coding for the antigen binding site. As such, almost all molecules can be antigens and the mechanism does not discriminate between self and non-self molecules.

B cells are APCs, like DCs and MPs. B cells internalize antigen bound to its BCR and, if it is a protein, will display peptides from the antigen on MHC II to activate and request help from CD4 helper T cells. A combination of molecular hugs between the TCR and the B cell’s MHC II-antigen peptide complex, CD40L and CD40 cell surface molecules, plus the cytokine conversation released by T cells will stimulate and promote differentiation of the B cell to undergo immunoglobulin class switching. As such, IgM and IgD defining immunoglobulin gene segments are deleted and the Fab defining portion is spliced to a Fc gene segment for IgG, IgE or IgA. Ultimately, B cells may differentiate into antibody factory plasma cells or memory cells. B cells also speak to other cells with regulatory, anti- or pro-inflammatory cytokine conversations.^39^^,^^40^

The conversation between B cells and T cells goes both ways. T cells promote immunoglobulin class switching and differentiation of B cells to memory or plasma cells. In return, B cells provide a very strong and antigen specific means for activating the T cell, especially during autoimmune responses.

## Initiation and interpretation of the cytokine conversation

Cells listen and respond to the different cytokines through cell surface receptors.^41^ There are several families of cytokine receptors. Expression of these receptors and subsequent intracellular signals determine which cells respond to the call for action and the consequences. Small molecules, ions, lipids, and chemically modified (phosphorylated or otherwise) proteins are the intracellular language that communicate the change requested by receptor activation at the cell surface.^42–44^ Cascades of serine and tyrosine kinases deliver much of the cytokine-receptor discourse.^45^^,^^46^ The signaling results in modification of structural elements, changes in metabolism, or activation of transcription factors within the nucleus to promote new protein synthesis, activate growth or alter the behavior of the cell and tissue.

## Conversation triggers: Danger signals-PAMPs and DAMPs

Danger signals that accompany trauma, infections and disease trigger innate and immune responses.^7^ Release of **DAMPs** upon cell death or injury communicate the need for a protective or defensive response and eventual repair.^47^ DAMPs are molecules that should not be found outside of the cell and include adenosine triphosphate (ATP) and heat shock proteins. **PAMPs** include microbial cell wall components, flagella, polysaccharides, other repetitive structures, and cytoplasmic nucleic acids. The presence of DNA and certain forms of RNA in the cytoplasm is abnormal and often indicates a viral infection of the cell. **Lipopolysaccharide (LPS)**, from gram negative bacteria, is endotoxin and a very potent PAMP. Receptors for PAMPs and DAMPs (**PAMPR and DAMPR**) are present on and in many different cells, but especially epithelial cells, MPs and DCs. These sensors include **Toll-like receptors (TLRs)** and other cell surface and cytoplasmic sensors.^48^^,^^49^

## Conversation triggers: antigens

B and T cell responses are initiated by antigen binding to BCRs on B cells or binding of antigenic peptide-MHC complexes to TCRs on T cells. However, the threshold for activation of these resting cells is very high to prevent spurious responses. To be considered real, the antigenic signal has to be sufficiently strong, sustained, and combined with other signals, as described below. Extensive descriptions and excellent figures describing T and B cell activation can be found in immunology textbooks.

To initiate a new conventional T cell immune response, a combination of signals is required to override the resting state of a naive T cell.^31^^,^^50^^,^^51^ Activated DCs provide the initial wake up call for naive T cells. The first activation signal comes from peptides presented within a cleft at the top of MHC I molecules to the TCRs on CD8 T cells or within MHC II molecules to TCRs on CD4 T cells.^15^ Hence, the response of conventional T cells is limited to proteins. The DC enwraps the T cell with its dendrites, interacting with many TCRs, and strengthens and lengthens the duration of the interaction with adhesion molecules. Activation requires that T cells receive sufficient antigenic stimuli through the TCR and a second signal through CD28 to override fail-safe **checkpoints.**^52^ A third signal comes from the cytokine conversation which determines the nature of the subsequent T cell response. Continued activation of T cells or reactivation of memory T cells can be performed by DCs or other APCs.

Initiation of a new B cell response also requires multiple signals.^53^^,^^54^ Crosslinking of BCRs by multivalent ligands plus TLR activation by PAMPs activate B cells and promote IgM production. IgM production is largely independent of T cells. Bacterial cell surface molecules (eg flagella, or capsular polysaccharides) are multivalent antigens as well as PAMPs. Activation is facilitated by the C3d component of complement attached to antigen and by cytokines including type 1 IFNs, B cell activating factor (BAFF) and a proliferation-inducing ligand (APRIL).^55^^,^^56^ Strong cytokine and receptor-ligand interactions can sometimes push the B cell to make IgG or IgA without T cell help.

T cells are usually required to promote immunoglobulin class switching to produce IgG, IgE, or IgA and for differentiation of B cells. B cells request help from CD4 T cells by internalizing antigen bound to their BCR and then proteolytically processing and presenting peptides from the antigen on MHC II to CD4 T cells. A T cell that recognizes the MHC II-peptide complex will reciprocate with activating signals of CD40L to bind to CD40 on B cells and with cytokines and other stimuli. This can initiate activation, growth, somatic mutation, genetic recombination for immunoglobulin class switching, survival and continued activation or subsequent differentiation of the B cell to become memory or plasma cells. Most of these reactions occur in the germinal centers of lymph nodes.

## Cytokine conversations

### Maintenance of immune balance, homeostasis and the status quo

**Figure 2:**
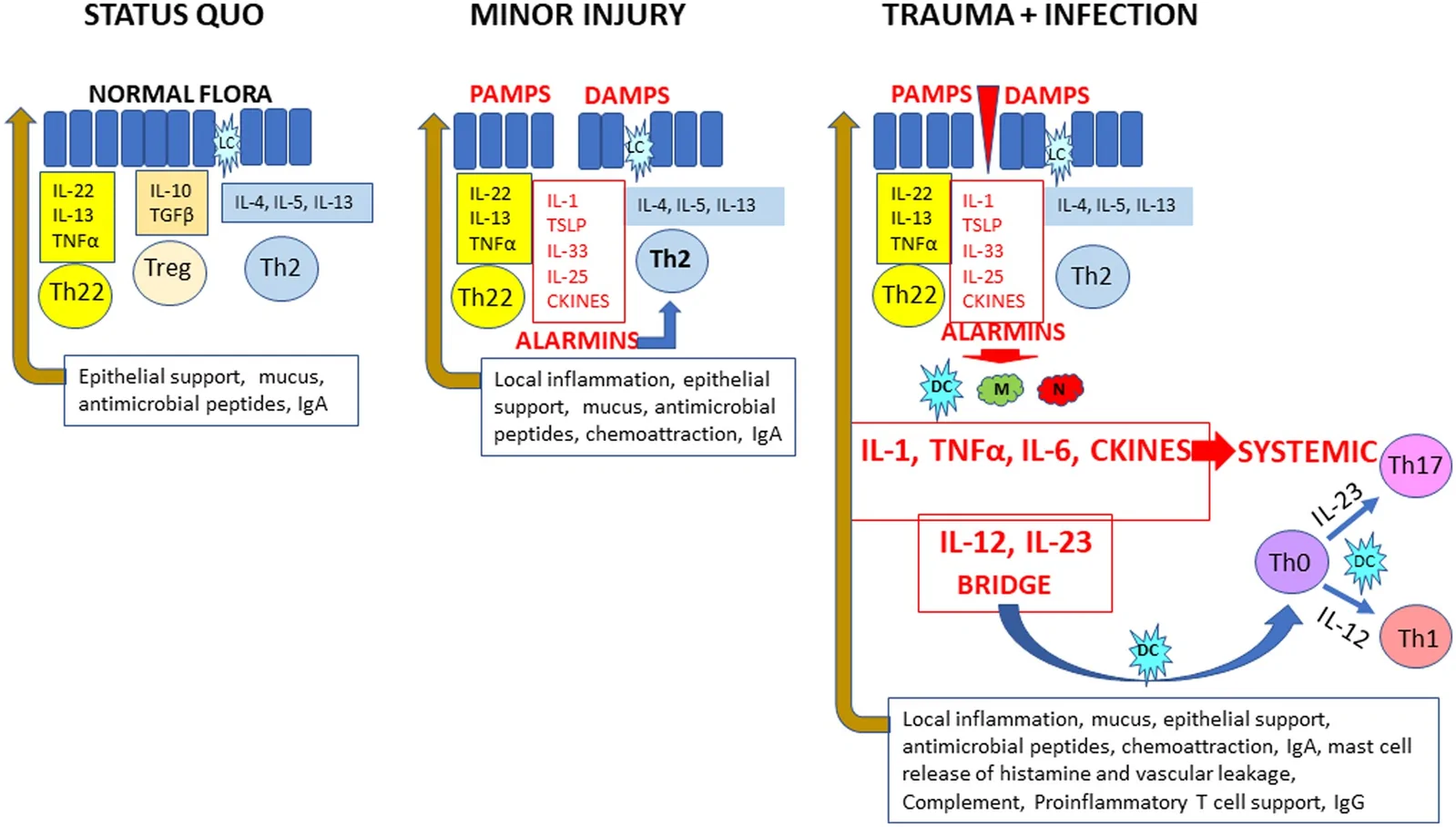
Cytokine conversations for status quo, routine minor injury, or a wound and infection. The status quo of the skin is maintained by innate responses that are reinforced by Treg, Th2, and Th22 conversations. Minor injury to the epithelial/keratinocyte barrier promotes release of alarmins, which activate local inflammation and reinforce Th2 responses. Trauma and infection causes release of alarmins and also activation of dendritic cells, macrophages and neutrophils which release acute phase cytokines to expand the local inflammation to include systemic antimicrobial support. DCs and LCs mature and travel to the lymph node to activate proinflammatory Th17 and Th1 conversationalists. DC, dendritic cells; LC, Langerhans cells; Th, CD4 T helper cell; IL, interleukin; PAMPs, pathogen associated molecular patterns; DAMPs, damage associated molecular patterns; CKINES, chemokines.

Maintenance of the status quo takes a considerable amount of effort, energy, communication and coordination. Status quo cytokine conversations maintain the size of immune cell populations and promote protections from day-to-day exposures to particles, proteins, and microbes at the openings of the body. All of this is done while limiting inflammation and promoting tolerance to self-proteins to ensure homeostasis and balance between inflammatory and anti-inflammatory responses (Fig. 2).

### Generation, expansion and mobilization

The bone marrow constantly generates new myeloid and lymphoid white blood cells to maintain normal levels of cells in response to different cytokine conversations for the different cell types.^57^^,^^58^  **Stem cell factor** (**SCF), IL-1, IL-3,** and other cytokines promote growth and maturation of lymphoid and myeloid stem and progenitor cells but expansion of lymphoid cells and myeloid cells are triggered by different stimuli and by different cytokine conversations.

**Lymphoid precursor growth** (B, T, ILC and NK cells) from the bone marrow is relatively constant and stimulated by conversations **that include IL-2 and IL-7**. Early in life, T cells generated in the bone marrow mature in the thymus but with the involution of the thymus later in life, T cell numbers expand from the established pool with the help of IL-7, IL-4 and IL-2.^59^ IL-7 can also be used to expand T cell numbers following a lymphopenia-inducing insult such as chemotherapy.^60^ In the periphery, lymphoid cell numbers increase in response to antigen stimulation plus **IL-1, IL-4, IL-2,** and other cytokines and stimuli.

**Myeloid cell growth** is stimulated by **GM-CSF, IL-3,** and other cytokines.^61–63^ Myeloid cell numbers expand when additional troops are needed to respond to infection or promote inflammation. These are triggered by acute phase cytokines and additional GM-CSF. **GM-CSF** plus **monocyte colony stimulating factor (M-CSF)** stimulate monocyte production and **GM-CSF** plus **granulocyte colony stimulating factor (G-CSF)** generate neutrophils. The increases in blood levels of neutrophils and neutrophil precursors is termed a “left shift.”^62^^,^^63^ Other myeloid cell types will potentially be produced in this response. GM-CSF is also an important part of Th1 and Th2 conversations.^62^^,^^63^ During inflammation, IL-3, promotes the growth and expansion of myeloid and lymphoid cells to facilitate the response.^64^

### Regulation, tolerance and maintaining immune balance

Recognition of self-molecules causing autoimmunity is one of the risks that accompanies an antigen detection system created by random recombination of TCR and BCR/immunoglobulin gene segments and subsequent somatic mutation of BCR/immunoglobulin genes. During the generation of new lymphocytes, most self-reactive T and B cells are eliminated by apoptosis within the thymus or bone marrow by mechanisms of **central tolerance**.^65^ Tregs escape the culling process and remain self-reactive and some autoimmune T and B cells slip through or are generated later. Self-reactive lymphocytes are controlled by mechanisms of **peripheral tolerance.**^52^^,^^66^ DCs, macrophages, ILCs, B and T cells facilitate peripheral tolerance with cell-cell interactions that use immune checkpoint and other molecules, by manipulation of metabolism and with tolerizing/regulatory cytokine conversations.^67^ These mechanisms also limit inflammation. Inflammation and T cell activation and proliferation are suppressed by cytokine conversations that include **IL-10, IL-35, and TGF-β**.

Tolerance and balance between pro-inflammatory and anti-inflammatory responses are especially important in the mucoepithelia of the respiratory and GI tracts to limit inappropriate inflammatory responses to common challenges, such as food and normal flora.^68^ Some intestinal Tregs express TCRs for food or microbe-related peptides and provide control by producing IL-10, TGF-β, or adenosine. In addition, regulatory reinforcements are generated by converting potential proinflammatory CD4 T cells into **peripherally derived Tregs** (**pTregs)**.^69^ Unconventional T cells, ILCs, and regulatory myeloid cells (MPs, DCs and even neutrophils) also promote tolerance but without antigen specificity. Inability to generate appropriate Tregs or pTregs can result in autoimmune or inflammatory diseases.^67^

### Antibody and Th2 stimulated immunity

**Figure 3:**
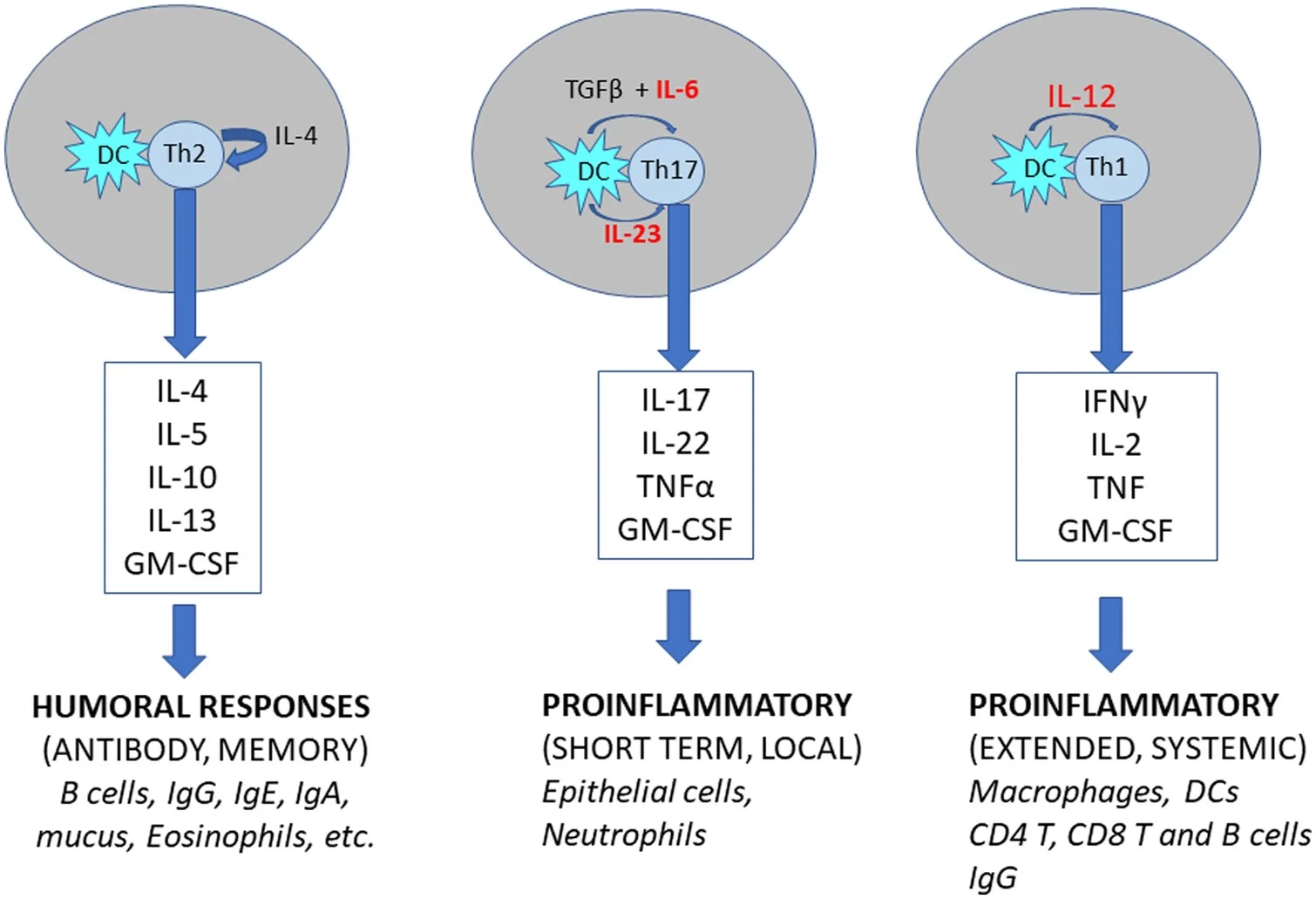
Cytokine conversations and response of Th2, Th17 and Th1 CD4 helper T cells. Dendritic cells activate antigen-specific Th responses and determine the nature of the response by the cytokines that they produce. Once initiated, other antigen presenting cells can restimulate the cells or activate memory cells. CMI, cell mediated immunity; DC, dendritic cell; GM-CSF, granulocyte macrophage colony stimulating factor; IL, interleukin; TNF, tumor necrosis factor.

**B cell differentiation** to produce IgG, IgE, or IgA is stimulated primarily by **IL-4, IL-5, IL-10, IL-13,** and **GM-CSF** by Th follicular cells in germinal centers of lymph nodes. The Thfs acquire transcription factors and cytokine conversations similar to Th1, Th2, Th17, and Treg cells^70^^,^^71^ (Fig. 3). IgG production is also promoted by IFN-γ produced by Th1 and Thf1 responses. Antibody can neutralize toxins, prevent microbial attachment, and opsonize debris for phagocyte clearance. The Th2 cytokines also promote mucin (mucus) production by goblet cells and activation of mast cells and eosinophils (IL-4, IL-5, IL-13).^72^^,^^73^

IgG, IgA, and mucin production are very important for protection from toxins and infection by extracellular pathogens. IgE, mucin production, and eosinophil activation are especially important for protective responses to parasitic worms.^73^

Th2 conversations dominate in some individuals and the imbalance can result in increased susceptibility for eczema and IgE-related allergies and asthma. A predilection for Th2 responses can also blunt the generation of Th1 conversations that can compromise protections against intracellular microbes, antiviral and antitumor responses. As such, a person prone to Th2 responses is at risk for more severe forms of tuberculosis, leprosy and leishmania.^74^

### Border maintenance

**Th22** conversations consisting of **IL-22, IL-13, and TNF-α** continuously support the health and function of skin and mucoepithelial membranes by promoting epithelial growth and health (IL-22), mucus production (IL-13) and antimicrobial peptide production (TNF-α).^75^ Upon microbial challenge, Th22 conversations are facilitated by IL-17 produced by proinflammatory Th17 T cells (see later). An excessive Th22 or excessive Th17 cytokine conversation can result in psoriasis of the skin.^76^

### Interferons: “GRRR”

**IFNs** are generated in response to PAMPs to activate intracellular protections from viral infection and to trigger inflammatory, antibody and cell mediated responses.^77^  **Type I IFNs include α, β, ε**.^78^^,^^79^ The most potent triggers are viral RNA, double stranded RNA and cytoplasmic DNA. Type I IFNs bind to the IFNAR receptor present on most cells and activate antiviral responses within the cell. Type I IFNs also promote MHC I and II expression, promote Th1 helper T cell responses, activate cytolytic NK and CD8 T cells and B cell activation. **Type II IFN** is IFN-γ. IFN-γ is produced by NK and T cells, is a potent activator of MPs and DCs, Th1 responses, and production of IgG. **IFN-λ, a Type III IFN**, also has antiviral activities.^80^

### Alarmins: “GRRR”

A conversation of **alarmin proteins** elicited by epithelial cells and local MPs provides rapid acknowledgement of danger from a wound, trauma or infection with minimal disruption of the status quo (see Fig. 2).^81^ These cytokines include **thymic stromal lymphopoietin (TSLP), IL-1α, IL-1β, IL-18, IL-33,** and **chemokines,** including **IL-8 (CXCL8).** The alarmin conversation activates responses from local DCs and MPs and promotes recruitment and entry of neutrophils and monocytes from the bloodstream but maintains a Th2 and Treg controlled environment. TSLP and IL-33 promote Th2 responses. Chronic activation of alarmin responses in the skin accompanies eczema and can predispose an individual to allergies due to the predilection for Th2 responses (“Atopic March”).^82^

**IL-1** exists as IL-1α and IL-1β which bind to the same surface receptor and induce the same responses.^83–86^ IL-1α is released upon damage to epithelial cells, fibroblasts and other cells while IL-1β is produced in response to an infectious or inflammatory challenge. Upon DAMP or PAMP stimulation, the **inflammasome** proteolytically activates and promotes release of IL-1β and IL-18 to support local inflammation and subsequent immune responses.^87^ IL-1α and IL-1β promote migration of neutrophils and monocytes into tissues, enhance inflammation and act as endogenous pyrogens (promote fever) as a potent acute phase cytokine.^83^^,^^86^

### Inflammatory responses: “GRRRRRRRRRRRRRRR”

#### Acute phase inflammatory responses

Upon damage or infection, local MPs and DCs, including Langerhans cells of the skin, respond to PAMPs and DAMPs by SHOUTING an **acute phase** cytokine conversation (**IL-1, TNF-α, IL-6 and chemokines)** that alerts and activates local and system-wide responses (see Fig. 2).^8–10^^,^^88^^,^^89^  *Many of the consequences of inflammation are initiated and mediated by acute phase cytokines.* Locally, this conversation promotes vasodilation (redness and heat), release of fluid containing antimicrobial substances into the affected site (swelling), recruitment and activation of neutrophils, mast cells, MPs and other cells, and triggers neurons (pain). These are the 4 main aspects of inflammation: rubor (redness), calor (heat), tumor (swelling), and dolor (pain). Systemically, the acute phase conversation puts the body into war mode. This includes elevated body temperature (fever) and a shift in proteins made by the liver’s protein factory to include acute phase proteins that support antimicrobial defenses and inflammation.^88^^,^^89^ These proteins include complement components and C-reactive protein.

**TNF-α,** with IL-1, are the main stimulators of inflammation. The ultimate consequence of TNF-α action is determined by the affected cell type and the influence of other stimuli (eg the cytokine conversation). TNF-α acts as a cell surface and soluble ligand to activate inflammatory pathways or apoptosis.^90^ As the name indicates, TNF-α can trigger death pathways in tumor and other cells. TNF-α binding to other receptors activates the nuclear factor-κB (NF-κB) transcription factor which promotes synthesis of many components of inflammation.^91^ TNF-α also stimulates histamine release from mast cells to promote fluid release from the vasculature.^92^ TNF-α and IL-1 inhibit erythropoietin production to reduce red blood cell production. Anemia of chronic inflammation (also known as anemia of chronic disease) may accompany chronic infection, autoimmunity or cancer and result from the combination of this inhibition and sequestration of iron by hepcidin, produced as an acute phase protein by the liver. TNF-α also promotes *cachexia*, the loss of appetite, weight, and muscle tone, which is why one of the early names for TNF-α was cachectin.

**IL-6** ^93^ ^,^ ^94^ and **IL-1**^83–86^ have many activities that support inflammation and the acute phase response.

#### Recruiting reinforcements with chemokines and complement

When local cells are overwhelmed by microbial and other challenges, they recruit neutrophil, monocyte and lymphocyte reinforcements with **chemokines**.^8–10^^,^^95^ Chemokines have four cysteine residues that determine a characteristic 3-dimensional shape. Different chemokines recruit different cells and elicit different responses within those cells. Expression of different chemokine receptors on T cell subsets direct the cells to where they are needed and to different regions of lymphoid tissue. **Chemokines stimulate conformational change within integrins on the vasculature that mark a path** that attracts and grabs white blood cells as they near the site and then promote their entry into the tissue.

When the vasculature is permeabilized, plasma components can leak into a wound or infection site. This allows access for the components of the **complement system (C′) (C1–C9)** and can enhance the inflammatory response.^96^^,^^97^ C′ consists of a proteolytic cascade of proteins which generate biologically active fragments (especially C3a, C5a) and a molecular drill for cell surfaces (C5–C9). Tissue surfaces that are disrupted by wounds or abrasion have the potential to activate all three complement pathways. Disrupted surfaces can activate the **alternate route,** microbial carbohydrates from normal flora can activate the **mannose route,** and immune complexes of IgM or IgG bound to antigen can activate the **classical route**. C3a and C5a are potent promoters of inflammation as attractants, like chemokines, and as activators of neutrophils and monocytes. Other products of C′ facilitate other responses.

#### Proinflammatory T cell cytokine conversations: “GRRRRRRRRRRRRRRRRR”

**Bridge cytokines to pro-inflammatory T cell responses** include **IL-23** and **IL-12.**^98^ When added to acute phase conversations generated by DCs or MPs, IL-12 and IL-23 activate Th1 or Th17 responses, respectively, to reinforce innate inflammatory responses. Both of these cytokines have a p40 subunit, but IL-23 also has a p19 and IL-12 has a p35 subunit.^99^

**Th17 conversations of IL-22, IL-17, TNF-α, and GM-CSF** reinforce local defenses at epithelial borders but also activate and extend the life span of neutrophils to promote inflammation (Figs. 2, 3).^27^^,^^31^^,^^32^ Th17 responses are triggered after APCs present antigen to T cells with a shout of IL-6 and IL-1 in addition to the status quo levels of TGF-β^100^ or with IL-23.^101^ High levels of TGF-β would inhibit Th17 responses.^102^ The duration of the acute inflammatory response is limited by the lifetime of IL-17 and neutrophils and the presence of specific antigens. However, memory Th17 cells are also elicited.^103^ Th17 activation of epithelial cells and neutrophils is very important for antibacterial and antifungal responses. Excessive or chronic Th17 responses exacerbate inflammatory diseases.^104^

**Th1 cytokine conversations of IFN-γ, IL-2, lymphotoxin (LT), TNF-α and -β, and GM-CSF** promote protections against intra- and extracellular infections and tumors that are more complete, systemic, and long-term than Th17 (Figs. 2, 3).^27^^,^^31^^,^^32^^,^^105^^,^^106^ IL-2 promotes lymphocyte and Treg growth, CD4 and CD8 T cell activation, and better immune memory.^106^ IFN-γ activation of monocytes and MPs converts them to ANGRY pro-inflammatory MPs with enhanced antimicrobial and inflammatory functions. TNF-α keeps them ANGRY.^107^ IFN-γ also enhances APC functions, promotes isotype class switching from IgM to IgG, but not IgE or IgA production, inhibits Th17 responses and facilitates CD8 T cell cytolytic responses to provide a more complete and lasting antimicrobial response.^107^

#### Prostaglandins and leukotrienes

Prostaglandins and leukotriene lipid mediators are produced by and influence many different cells.^108^^,^^109^ Prostaglandins are defined by their structure and ability to activate or suppress immune and inflammatory responses. The responses are dependent upon cellular expression of their receptors. Proinflammatory cytokines that induce or elicit the Th1 or Th17 responses promote expression of different prostaglandin receptors than Th2 or regulatory Treg cytokines to allow the prostaglandins to enhance the response. Prostaglandin E2 (PGE2), for example, can promote the classic signs of inflammation: redness, swelling, and pain but can also promote anti-inflammatory responses and regulation.^110^ Receptors for PGE2 are present on MPs, DCs and T and B lymphocytes. PGE2 can modulate the entire progression of inflammation depending upon which cells are expressing the receptors.

## Consequences of inappropriate cytokine conversations

When cytokine conversations are excessively loud, inappropriate, extended (chronic), uncontrolled or disorganized they can lead to disorder, dysfunction and disease. For example, excessive Th22 or Th17 responses in the skin can promote psoriasis and excessive Th2 responses promote allergies, asthma and eczema. Insufficient regulatory responses allow autoimmune and inflammatory diseases. Knowing the type of conversation that is driving a disease allows appropriate selection of therapy. Monoclonal antibodies can neutralize key cytokines or block their interaction with receptors and small molecular inhibitors of receptor activated Janus kinases can block cytokine activated pathways that mediate the disease response.^3^

## Summary

Maintenance of the status quo, the initial response to tissue damage and infection, and the subsequent systemic alert that reinforces immune protections require considerable communication and coordination. Although individual cytokines have their own activities, it is the cytokine conversation that communicates and provides the coordination necessary for the response. The conversations can be summarized as those that maintain the status quo: Th22, Treg, and Th2; those that initiate local inflammation: alarmins and acute phase; those that recruit systemic support: acute phase and dendritic cell associated bridge cytokines; and proinflammatory responses that reinforce local and systemic protections: Th17 and Th1. Inappropriate or excessive cytokine conversations cause disease. Ablation or inhibition of the cytokines within disease driving conversations can be therapeutic but can increase susceptibility to certain microbes.

## Data Availability

All data are available through the references. This review contains no new data.
